# The readiness to use quantified self-technology: A case of diabetic patients from a hospital in Bulawayo, Zimbabwe

**DOI:** 10.1177/20552076251376286

**Published:** 2025-10-28

**Authors:** Belinda Mutunhu Ndlovu, Baldreck Chipangura, Shawren Singh

**Affiliations:** School of Computing, 56866 University of South Africa, Pretoria, South Africa

**Keywords:** Technology readiness, quantified self technology, diabetes, adoption, diabetes monitoring

## Abstract

**Objective:**

Quantified self-technology (QST) can potentially improve the monitoring and management of diabetes. Despite its potential, its uptake by diabetics in developing countries is notably low. This study investigated the underlying factors influencing the readiness of diabetics to adopt QST. The research question posed was, ‘What are the factors that inform the readiness of diabetics to adopt QST in developing countries?’.

**Methods:**

Semi-structured interviews were employed to collect qualitative data from a sample of 35 participants aged between 18 and 65 from a hospital in the city of Bulawayo, Zimbabwe. The participants were selected through self-selection sampling technique. Data was analysed using Braun and Clarke's thematic data analysis.

**Results:**

Three factors were found to drive the readiness to adopt QST and they are awareness, optimism and social support. Conversely, two factors were found to inhibit the readiness to adopt QST and they are insecurity and challenges.

**Conclusion:**

The findings suggest that patients are more likely to be ready to use QST if they are aware of the technology, optimistic and have adequate social support from family and community. Moreover, readiness can be enhanced if the patients’ challenges are addressed and insecurities are demystified. The implications of QST on diabetics are that it enhances self-monitoring of diabetes, which in turn improves preventative health and reduces hospital visits by diabetics. The implication on health policy is that adoption of QST reduces health cost of treating diabetes, hence, policies should provide enabling environment for the use of QST technologies by diabetics.

## Introduction

In less developed countries, diabetic patients manage their ailments by relying on traditional methods such as infrequent visits to the hospital, the use of glucose meters, and manual recordkeeping.^1,2^ The methods are less effective in managing diabetes because they are prone to errors, recollection bias, and do not provide real-time data.^3,4^ In retrospect, some traditional methods were found not to empower patients to proactively participate in their own healthcare.^5^ Whilst challenges associated with traditional monitoring are documented, the potential benefits of quantified self-technology (QST) can potentially overcome the challenges of diabetes management.^4,6^

QST refers to the utilisation of technology to gather, analyse, and interpret physiological and biological data for the purpose of self-awareness and enhancement across several aspects of one's life.^7–9^ QST is also referred to as self-tracking, self-monitoring or lifelogging^10^ and it utilises tools such as wearable devices and mobile applications to track, collect and monitor personal health.^7,11,12^

The use of QST facilitates self-monitoring of blood glucose levels and further promotes healthy living by rewarding exercising, weight management, adherence to prescribed medication routines and a healthy diet.^13,14^ Furthermore, QST provides personalised feedback and medical recommendations on essential metrics that can help alleviate blood sugar levels. The technology provides an option for sharing data with healthcare professionals and for patients to receive online consultations.^15^ By automating data collection and offering accessible visualisations, QST enables patients to attain a comprehensive picture of their diabetes condition and to make educated self-management choices.^11^

To a greater extent, the benefits of QST have been embraced in developed nations, whilst developing nations are lagging.^15^ Some literature has attributed this status quo to the challenges of the digital divide, which include digital illiteracy, inadequate technological infrastructure, diverse cultural perceptions, and financial constraints.^16^ These impediments may affect the readiness of diabetic patients to adopt QST in developing countries.^17^ Even though developing countries were found lacking in QST adoption, some research found that information technologies have permeated all facets of the lives of people in developing countries.^18,19^ The challenges observed in the adoption of QST can be unique. Therefore, there is a disparity between general technology adoption and the adoption of QST in developing countries. Hence, this research investigates the readiness of diabetic patients to adopt QST for managing and monitoring diabetes. This translates into the following research question: What are the factors that inform the readiness of diabetic patients to adopt QST in developing countries? The scope of this study is limited to users who make use of QST applications that run on mobile devices such as smartphones and wrist watches.

## Literature review

The readiness to adopt and use QST for monitoring and managing diabetes can be understood through the lenses of several theories. This study focuses on the constructs of the Diffusion of Innovation theory (DOI),^20^ Technology Readiness and Acceptance Model (TRAM)^21^ and the Technology Readiness Index (TRI) model.^22^

DOI explains how new innovation gets spread and accepted among members of a community.^20^ The attributes of innovation were characterised as relative advantage, compatibility, complexity, trialability, and observability. One important aspect of diffusion of innovation is the time at which the innovation is accepted by community members, who were classified into innovators, early adopters, early majority, late majority, and laggards. In practice, the theory has been applied to investigate the readiness of health workers to adopt new technologies in Mauritius^23^ and Germany.^24^ The theory was also applied in predicting the readiness of teachers to adopt teaching technologies in Israel.^25^ In the context of DOI, there is a study^25^ that asserted that user knowledge and attitude toward a technology influence their readiness to adopt that technology.

The TRI model explains an individual's desire to adopt a new technology in life or at work.^22^ The four constructs of the model are optimism, innovativeness, discomfort, and insecurity. The model has been applied in different contexts and varied results were obtained. In India, it was found that insecurity affected the readiness of users to adopt technology in war-torn areas.^26^ In a study carried out in Chile, it was found that age and culture moderated the readiness to adopt technology.^27^ In South Africa, it was found that optimism and innovativeness significantly affected readiness to adopt asset-tracking software in disadvantaged schools.^28^ However, in developed countries, a study carried out in Finland found that age moderated optimism, innovation and discomfort.^29^

TRAM theorised that constructs of TRI are the antecedents of both perceived usefulness and ease of use, which both, in turn, determine the user's intention to use e-services.^21^ Basically, the model combined the four constructs of TR (optimism, innovativeness, discomfort, and insecurity) and two constructs of TAM (perceived ease of use and perceived usefulness). The model has been tested in different contexts and varied results have been obtained. Results from a study carried out in Jordan found that perceived usefulness and perceived ease of use significantly determined customers’ intention to continue using self-service kiosks.^30^ In another study that investigated the readiness to use wearable technologies in South Africa, optimism and eagerness significantly predicted acceptance to use the technology, whilst discomfort was insignificant.^31^

From the constructs of the theories discussed, we learned that DOI characterised technology adoption with constructs: relative advantage, compatibility, complexity, trialability, and observability.^20^ The TRAM extended the TRI model and both have four common constructs: optimism, innovativeness, discomfort and insecurity.^21,22^ The TRAM has two more constructs, perceived ease of use and perceived usefulness.

Comparative analysis of the DOI versus TRI and TRA constructs revealed that the constructs can be grouped into drivers and inhibitors of readiness to adopt a technology. The drivers for DOI are relative advantage, compatibility, trialability and observability, whilst for TRI and TRA are optimism, innovativeness, perceived ease of use and perceived usefulness.

The constructs relative advantage and optimism are aligned. Relative advantage pertains to the degree to which an innovation is perceived as being better than an idea it supersedes.^20^ The advantages can be social or economic. From the TRI and TRAM models, optimism pertains to positive perceptions that an innovation improves the lives of the users.^22^

The constructs trialability and innovativeness are aligned with each other. From a DOI perspective, early adopters are those who try new technology to evaluate if it meets their needs.^20^ Similarly, the innovativeness construct of TRI and TRAM pertains to the evaluation of innovation by pioneers to see if it meets their needs.^22^ There is a study^32^ that argued that the two constructs are similar because the users who try new innovations are the innovators or early adopters. These are the users who are aware that such a technology exists.

The constructs of complexity and discomfort are aligned with each other. Complexity is the perceived difficulty in understanding and using technology,^20^ which is similar in meaning to discomfort.^22^ Complexity negatively affects readiness to adopt a technology because it was found to cause anxiety.^33^ Factors that cause technological anxiety include low efficacy, which can be a result of low digital literacy.^34^ If users perceive a technology as difficult to use, complex, or uncomfortable, that indicates their lack of readiness to adopt it.^21^ Furthermore, if users are not confident in using a technology, they can perceive it as insecure because they are sceptical that the technology will not work as expected.^32^ Aligned with discomfort is insecurity when using online technologies, which can be caused by fear of cyber risks such as data breaches and identity theft.^35^

From DOI perspectives, compatibility is how a technology consistently supports the lived experiences of users.^36^ With respect to TRAM, compatibility is aligned with perceived usefulness, which pertains to how the users perceive technology as useful in supporting their needs.^21^ If a technology is useful or compatible with existing life, then it will have an impact on the social life of users. Evidence from research revealed that technologies that have tangible results receive social support from the community.^37,38^ There is a study^37^ that found that technology users who receive social support from friends and family are ready to use technology. This aligns with the results of a study that found that motivational, emotional and financial support increases readiness to adopt technology.^38^ Conversely, a lack of support was found to cause disengagement, especially among the elderly.^39^

## Methodology

### Study setting

This qualitative study leveraged an interpretivist research paradigm and qualitative data were collected from a single public hospital in the Bulawayo city, Zimbabwe. The hospital was selected because it provides representative services that can be found at any other hospital in the country. Furthermore, the demographics of diabetics who visit the hospital provide a representative sample of people with diabetes in the country in terms of gender, economic status and academic attainment. Hence, the hospital from which the data was collected is a typical case of a hospital in Zimbabwe.

### Sampling and participant recruitment

A total of 35 diabetics who receive treatment at a hospital in Bulawayo city were recruited to participate in the study through self-selection sampling, which is a non-probabilistic sampling technique.^40,41^ To recruit participants, the hospital was approached to advertise and distribute a participant information sheet to diabetic patients who visit the hospital for routine check-ups. The participant information sheet introduced the concept of QST in layman terms and outlined the objective of the study. Some advantages of QST in managing diabetes were outlined using a neutral language to minimise biases and over expectations by participants. The participant information sheet provided contact details that included telephone number and email address of the data collection researcher for queries.

The participant information sheet stipulated the inclusion and exclusion criteria. To be included in the study, a participant should have been diagnosed with type 2 diabetes for at least one year, be of any gender, be between the ages of 18 to 65 years, have access to an internet-enabled smart phone device, and reside in Bulawayo city. Therefore, any potential participants who did not meet the stipulated criteria were excluded from the study.

The participants who met the criteria and were willing to participate in the study completed the participant information sheet. A total of 42 forms were returned to the hospital, of which, two forms were incomplete and were removed from the sample as spoilt forms. There were three forms with telephone numbers that were not reachable, and the forms were removed if the telephone failed to get through after three attempts at different intervals. To complete the recruitment process, the researcher contacted all the participants who had correctly completed the participation information sheet through telephone calls for introductions and to confirm that all participants wanted to participate. The researcher explicitly communicated that participation was voluntary, and no compensation was going to be provided in any form. A total of two participants anticipated that they would get some material benefits or compensation and were removed from the study.

Consequently, 35 diabetic patients were recruited to participate in the study. The final sample size was reached through removing participants who did not meet the inclusion criteria. However, research suggests that there is no universally accepted sample size for qualitative research, with some suggesting a sample size between 10 and 40 participants.^42–44^ The overall demographic characteristics of participants are presented in Table 1.

**Table 1.** Demographic distribution of participants.

	Age
Age range	18–35	36–45	46–65
Sample proportion of number of diabetics	10	12	13
	Gender
Gender type	Male	Female
Sample proportion	13	22
	Highest educational level
Highest level of education	tertiary	secondary	primary
Frequency	25	8	2
	Years with diabetes
Years with diabetes	More than 1 year	2–5years	6–10 yrs	More than 10 years
Frequency	4	12	14	5

### Data collection

The interview protocol had six open-ended questions derived from literature analysis. The development of the interview questions was informed by the findings of literature analysis. Pilot testing was done with five participants to test the data collection instrument. Incites from pilot test were used to improve the clarity of the interview questions. Table 2 matches the questions with the constructs of theories that inform the readiness for technology adoption discussed in the literature.

**Table 2.** Interview questions.

Question	Construct
1. Which health technologies for managing diabetes do you know of, or have you used?	Awareness
2. What do you think are the benefits of using health technologies for managing diabetes?	Relative advantage
3. Do you think the use of health technologies in managing diabetes aligns with the way you live your daily life?	Compatibility
4. Which challenges do you think will affect you when you use health technologies for managing diabetes?	Discomfort
5. What are your fears with respect to the use of health technologies in managing diabetes?	Insecurity
6. What kind of support do you think you will require to help you be ready to utilise technology?	Support

Face-to-face interviews were conducted from May 2024 to July 2024 in the participant's selected location, offering a comfortable and confidential environment. Each participant signed an informed consent. The interview duration was between 45 and 60 min, which provided participants with sufficient time to articulate their opinions and experiences. All interviews were audio-recorded with the participant's consent and transcribed verbatim. To confirm the accuracy of data captured during the interviews, participants were provided with the interview transcripts to verify accuracy of transcriptions. Four individuals accepted to review the transcripts but did not provide any feedback.

### Data analysis

Data analysis was qualitative and employed the Braun and Clarke thematic analysis method.^45^ Atlas.ti, a computer-assisted qualitative data analysis software was used. Data analysis encompassed six phases. Phase 1, researchers familiarised themselves with the interviews by transcribing and revising the transcripts to note key ideas. Phase 2, the researchers generated the preliminary codes by reading the whole data set. Two coders generated the initial codes independently of each other to ensure intercoder reliability. All authors held iterative debriefing meetings to agree on codes throughout all phases. Phase 3, the researchers collated the generated codes from Phase 2 into themes. All the codes belonging to a theme were gathered from all the participants’ scripts. In Phase 4, the generated themes were reviewed to check if the codes were grouped and aligned correctly. In Phase 5, the identified themes were reviewed and themes with the same meaning were merged and renamed to create a final set of themes. The final stage of thematic analysis involved writing up the results as presented in the results section which are in accordance with the consolidated standards for reporting qualitative research checklist.

Data saturation was reached after analysing 16 scripts, however, data analysis was not stopped at saturation to have broader views of participants. This approach was essential for maximising data use and getting a wide array of perspectives to improve the credibility of the findings. Thus, all the 35 scripts were analysed in this study. Moreover, credibility was enhanced by building rapport with the research participants. A reflective journal was composed immediately after each interview. To ensure conformability, peer debriefing meetings were held, and member checking was done after the transcription of an interview. Dependability was upheld by an audit trail to document all the procedures, methods, and decisions taken by the researchers. Lastly, transferability was upheld by providing rich, thick, detailed descriptions through quotations from research participants from a health resource context.

### Ethical considerations

This study received ethical clearance from the University Research and Ethics Committee Ref (2023/CAES_HREC/1791). The university ethics policies, hospital code of ethics and data protection policies informed data collection. Additionally, participants signed the informed consent to participate in the interview. Oral consent was also sought from participants before recording. Participation was voluntary, and no financial incentives were paid or promised to participants.

## Results

The results of the data analysis uncovered five themes that can explain the readiness of diabetic patients to adopt QST for monitoring and managing diabetes. The five themes are: Awareness of technology, Social support, Optimism, Challenges, and insecurity. Table 3 presents the themes and their sub-themes.

**Table 3.** Themes and subthemes.

Theme	Subthemes
Theme 1: Awareness of technology	Lack of exposure
Digital illiteracy
Medical practitioners not sharing information
Prior experience
Theme 2: Social support	Family support
Social group support
Theme 3: Optimism	Enthusiasm and eagerness to learn
Theme4: Challenges	Compatible smartphone ownership
Internet access
Power supply challenges
Technology anxiety
Theme5: Insecurity	Data theft
Identity theft

### Theme 1: Awareness of technology

Awareness entails knowing that a technology exists. Data analysis uncovered that the sampled diabetic patients had varied knowledge of QSTs. Twenty-four out of 35 participants pleaded ignorance of the technology, and the remaining participants were knowledgeable about QST. Three factors were found to influence the lack of awareness of QST, and they are a lack of exposure, digital illiteracy, and medical practitioners not sharing information with patients.

Lack of awareness of QST was attributed to the lack of health education provided to the diabetics neither at health facilities nor in the media, for example, in newspapers, radio or television. Furthermore, the elderly participants who were ignorant of QST associated mobile-based applications with younger generations or educated people. This is an issue of digital illiteracy amongst elderly people. A few participants pleaded that this was their first time to hear about QST technology and blamed it on their low education levels. In this respect, Participants P2 and P4 said:

I have not used any technologies to manage my diabetes. I have never seen any advert or awareness programs on television or newspapers or even in the hospital. (P2)

I also thought these technologies are for educated people and I was very wrong. I wish I was that educated and maybe associated with learned people, and they would have referred me to this technology. (P4)

The lack of awareness was partially blamed on healthcare practitioners who were accused of concealing information about QST from diabetic patients. Participants were of the view that their practitioners were supposed to introduce them to the technology when they were diagnosed with diabetes. Some participants were suspicious that the practitioners were concealing information so that the patients would depend on them for routine check-ups. In this respect, Participant P12 said:

So, when l first started using the medical apps, the doctor wasn’t happy but l realised that these doctors are after money. This is because for every appointment I book I pay a consultation fee. (P12)

There were 11 participants who were knowledgeable about QST. The participants attributed their awareness to prior experience with using similar technologies, for example, applications used for fitness tracking. Thus, the participants were able to identify QST applications, for example, Home Workout App for fitness tracking, and Dr Blood for measuring blood pressure. The data suggested that prior experience with health-related applications improved participants’ readiness to embrace new technology. In this respect, the participants P20 and P21 said:

I was already familiar with a few apps for monitoring health, especially when it comes to managing my diabetes. I’ve been using a glucose monitoring device for years that connects with my smartphone. This allows me to easily track my blood sugar levels throughout the day and spot any concerning trends…. (P21)

I use the MyFitnessPal app for general nutrition and Google Fit for tracking my physical activity. I have used MyFitnessPal for about six months and Google Fit for about a year…. (P20)

There were participants who got to know about QST through referrals from other people who had used similar apps, their children, or their ability to carry out personal research about their health and technology trends. In this respect, the participants P21 and P34 said:

I have observed a rising acceptance of digital health tools in my larger community. It seems like more and more people in my social circle are tracking their own health indicators with wearables or similar apps…. (P21)

I have a qualification in computers, so I usually meet these things when I am researching…. (P34)

### Theme 2: Social support

The readiness of some of the participants to use QST resulted from the support that they received from their family and community members. The elderly people indicated that they depended on their younger family members to use mobile technologies. For example, an elderly woman (P32) indicated that her children helped her with installing applications on her mobile phone. Respectively, a middle-aged woman (P2) indicated that her children would remind her to take medication if her phone sent reminders. In this respect, the participants said:

My kids are quite amazing because sometimes I am not where my phone is and then a notification pops up, reminding me to take my medication. If they are close to the phone, they know they should shout “Mama, please come.” (P2).

Everything on my phone was done by my daughter, diabetes has also affected my right eyesight. Although I can download and install apps on my own, I struggle to read the text font on my phone, hence, my daughter helps me with app installation. (P32)

Financial assistance was identified as a crucial readiness factor that came from family support. There were participants who received money donations from family members to cover data bundles (broadband) and app subscription expenses. Financial support is an essential readiness factor because it opens access to QST technologies, hence promoting a seamless incorporation into everyday life. In this respect, participants P9 and P20 said:

My siblings help me with buying data bundles, which I need. My aunts and cousins also help by paying or topping up for my subscriptions. (P9)

My husband has been supportive in using the app for monitoring diabetes because he makes sure that l always has data bundles (broadband) and pays for the monthly subscriptions for reminders to take medication. (P20)

Social connections through different support groups provided emotional support, dissemination of knowledge, and encouragement. In the social support groups, individuals shared experiences and knowledge about life in general and chronic health challenges. Data analysis uncovered that social media platforms such as WhatsApp facilitated dialogue on the social groups. Stigma also emanated from the social groups toward technology utilisation in health management, especially from members of some church denominations who opposed the use of technology in health. This contradiction shows possible obstacles that may emerge in social support networks, where conventional perspectives may clash with modern health methods. In this respect, participants P8 and P25 said:

… at first some of my friends were against the whole idea of using this technology, thinking that l was not going to be able to manage the condition well. However, through shared experiences and learning more about the technology, people became more positive about it. (P8)

My pastors at church don’t support the use of technology and taking medications for diseases because they say they can be cured by prayer. They say that these are spiritual diseases. At church, they distrust the technology… (P25)

### Theme 3: Optimism

The findings revealed that some participants had a proactive and optimistic disposition towards technology. The participants were dependent on mobile phone applications for managing their everyday activities, for example, tracking daily physical activities. The participants showed enthusiasm in embracing new health technologies with the zeal to learn, which underscored a positive attitude. Amongst the participants were early adopters of QST, who acknowledged that the applications empowered them and gave them autonomy to self-manage their ailments. The findings indicate that eagerness and enthusiasm are driven by curiosity, the pursuit of convenience, or technological advantages. The results suggest that the optimistic participants were ready to adopt QST. In this respect, participants P11 and P14 said:

I cannot imagine my life without my phone … I use my phone to do everything from monitoring my sugar levels to counting steps, my daily routines, etc. Technology makes monitoring one's health so easy and it's so easy to use. (P11)

Learning new things is fun. The use of the app is a great idea, and I am the pioneer in using this app in my family. My family got so interested in knowing more about the app that I continued using it because they wanted to learn more about it. (P14)

### Theme 4: Challenges

The first obstacle was not having access to a smartphone handset. One of the minimum requirements of a QST is a smartphone with an internet connection. Some participants contemplated upgrading their smartphones to meet the requirements of QST, which demonstrated how obsolete technology can discourage the adoption of a new technology. One participant emphasised that without a multifunctional device, the advantages of self-tracking remain unexploited. Consequently, not affording a compatible smartphone can impede an individual's readiness to adopt self-tracking technology. In this respect, participants P7 and P25 said:

Firstly, I had to have a gadget that connects to the internet, which is a smartphone. Secondly, I needed to have access to Wi-Fi, which requires subscription. (P25)

It was necessary for me to ensure I had a good smartphone with long battery life, a big screen, and storage. (P7)

The second obstacle was intermittent electrical supply, which was observed to affect the internet connection and the lifespan of the mobile device battery. Because power cuts were reported to be frequent and lasting many hours in Zimbabwe, some participants indicated that they relied on alternative sources of power such as power banks and solar panels. The negative effects of intermittent electrical power supply were echoed by most participants, which highlighted the gravity of the challenge to the readiness to adopt QST. In this respect, participants P1 and P19 said:

A power bank will be ideal, especially when there is no electricity, just yesterday the hospital had no electricity from early morning to around 6 pm which may disturb the use of the app. A power bank may also be useful when I am traveling…. (P19)

We spend most of the time without electricity, so I must make sure my phone is fully charged. When my phone is fully charged, I will not miss any updates on my app as well as reminders or alerts to take medication. (P1)

The third obstacle hindering readiness to adopt QST was the lack of finances to pay for the associated digital expenses. Most participants were concerned about their inability to fund and sustain internet-based health technologies. The identified expenses were the cost of internet connection (broadband), new devices, and subscription fees. Explicitly, 20 participants indicated that they had financial limitations and could not sustain paying subscription fees for health applications. The financial burden highlighted by the participants suggested a lack of financial readiness to adopt QST. In this respect, participants P9 and P14 said:

The other support that I need is the money to pay for the subscriptions. After doing a cost and benefit analysis, I’ve realised that paying the yearly subscriptions at once is cheaper than paying for the monthly subscription. (P9)

I would love to have several things to effectively use this app, such as prepaid Wi-Fi, preferably for the whole month. This might save me from worrying about money to purchase data bundles. (P14)

The fourth obstacle was technological anxiety. The results uncovered that elderly participants’ anxiety was caused by the phobia of continuously asking for help to use mobile applications. They were not ready to continuously depend on young people, which made them frustrated, rendering the technology seem unattainable. Some regarded the never-ending need for monitoring and receiving updates as invasive and disturbing. Continued involvement with health monitoring was perceived as dominating their lives with diabetes management. Hence, this can have a negative consequence on the readiness to adopt QST. In this respect, participants P19 and P32 said:

I dislike that I need to be constantly checking for notifications and updates on my phone. I find it unsettling to be scrutinised for each minute. … It's just too much. I feel uncomfortable and having no control over my life. (P19)

I no longer sleep properly because I am now just too anxious with each coming day that I must do this and that …. Measuring, reminders, eating health … Worse when I see that that my sugar is going up, get troubled. (P32)

### Theme 5: Insecurity

The first security concern was identity theft. Many participants were concerned about identity theft and abuse of their personal information, which could result in cyber reputational damage. Furthermore, some feared that if their identities were stolen, criminals would use their identities for financial transactions, which may result in financial loss. These challenges exacerbated anxiety, affecting readiness to adopt QST. In this respect, participant P35 said:

My identity can be stolen, and people can commit crimes pretending to be me, and I will end up landing myself in jail or being blacklisted from getting better job opportunities. (P35)

The second security concern was data theft. The participants feared that data collected by QST could be sold to data brokers, advertising agencies, or used by hospitals for financial profit through deceptive practices to solicit donations. Participants perceived their health data as confidential and feared that data breaches could expose their ailments to a broader audience. Coupled with unauthorised data sharing on social media, participants were concerned about the risk of social stigma. Furthermore, the possible exploitation of participants’ medical history by insurance companies was also a concern, with participants being worried about being classified as higher risk. This demonstrates that privacy violations can diminish trust in self-tracking applications. In this respect, participants P5 and P17 said

I would not like my information to be used by third parties without my permission, for example, my data can be stolen and misused to do unethical things which might drag my name into jail. (P17)

I would want to know who else has access to my data, … If my data is accessed by an insurance company, it will be a worry to me, it will be more like they are adding me to their death statistics, a countdown of my living days. (P5)

## Discussion

This section answers the research question, ‘What are the factors that inform the readiness of diabetic patients to adopt QST in developing countries’. The results of the data analysis uncovered five themes that determine the readiness to adopt QST, and they are: Awareness of technology, Optimism, Social support, Challenges and Insecurity.

Data analysis results found that most participants (24/35) were not aware of QST. Lack of awareness was found to be influenced by factors such as lack of health education, the perception that mobile-based apps are for youngsters, and medical practitioners not introducing such apps to patients. These factors were dominant amongst the elderly participants. On the other hand, 11/35 participants were aware of QST technologies. Their awareness resulted from prior experience or being introduced by friends or family members to QST applications. Most of the participants who were aware of QST were young participants. The findings of this study suggest that awareness of QST amongst the elderly can be improved by providing health education. The finding resonates with literature that found that awareness is often lacking, especially among certain demographic groups and geographic areas, for example, third world countries.^39,46^ This study suggests that QST can be introduced to patients through medical practitioners, family members and social groups. This aligns with the findings of Park et al.,^47^ who concluded that a lack of awareness of health technologies by patients can be addressed by providing health education. Furthermore, there is a study^48^ that argued that health practitioners should be instrumental in proactively sharing information about health technologies, their advantages and disadvantages with their patients.

The preconceived perception by the elderly that mobile-based apps are for youngsters should be changed. This aligns with Kenny and Connolly,^49^ who argued that there is a need to raise awareness and make technology inclusive for everyone, without limiting it to young people, because the elderly can also benefit. The results of this study revealed that the readiness of diabetic patients to use QST is reinforced by family and community support. Positive and negative social support instances that affect the readiness to adopt QST for managing diabetes were uncovered. To enhance positive social support, family and community members can enhance the use of QST by providing emotional and financial support. The findings of our study resonate with studies that found that patients who receive support and motivation from family and friends are ready to utilise health technologies.^37,38^ Ultimately, the active participation of family members as agencies of social support fosters an atmosphere that encourages diabetics to effectively utilise technology for enhanced health practices.^38,50^ To diminish negativity associated with the stigmatisation of the use of QST in monitoring and managing diabetes, communities can be provided with technology readiness educational programmes designed to offer constructive assistance. These findings align with literature that found that not all types of social groups are beneficial, as some can unintentionally present adverse effects.^51,52^ Negative criticism or unsolicited advice can diminish an individual's confidence and motivation, fostering feelings of inadequacy.^53,54^ Hence, to improve readiness to adopt QST, family and community should positively support diabetic patients.

The optimism to use QST was driven by eagerness, enthusiasm, curiosity and the pursuit of convenience. Curiosity drove the patients to discover QST, and their eagerness motivated them to learn how to use the technology to reap the benefits. Readiness to discover new technologies characterised the innovators amongst the participants. The results suggest that optimistic patients are ready to adopt QST. For patients to be optimistic, they must be positive, active users of technology and motivated to reap the potential health benefits of QST. The findings correspond with the results of a study that found that positivity improves individuals’ interaction with health technologies.^55^ The findings of this study build on the findings of a study^56^ which asserted that a knowledgeable patient is more inclined to adopt and utilise health innovations efficiently. However, there is a study^57^ that cautioned that exaggerated optimism due to disillusionment may underestimate the challenges of incorporating technology into daily routines. It is essential to acknowledge that not every participant exhibited positive optimism about QST, which could negatively affect readiness. This calls for a personalised approach that addresses varied attitudes towards technology adoption. This can be achieved by ensuring that optimism is bolstered by providing training and support to fulfil user expectations.

There are four challenges that were uncovered from data analysis that inhibit the readiness to adopt QST. Firstly, readiness to use QST for monitoring diabetes can be inhibited by a device that a user owns. Diabetics may require particular smartphone models to effectively deploy QST because some operating systems are not compatible.^58,59^ The implications are that users who have incompatible devices are automatically not ready to use QST, which is an obstacle to potential users who cannot afford QST compatible smartphones. This finding corresponds with the findings of a study^60^ which underscored that the availability of appropriate devices is essential for efficient interaction with health technologies. Secondly, the readiness to use QST can be inhibited if users cannot afford to pay for broadband, which can result in temporary internet disconnection. This aligns with the findings of a study^61^ that indicated that inadequate internet access challenges technology use by diabetic patients in low-resourced communities. Lack of connectivity hinders real-time data collection and monitoring, which ultimately affects diabetes management.^62^ Thirdly, intermittent electrical power supply inhibits the readiness to use QST. Intermittent electrical power supply is a challenge that was found to be unique to developing countries and profoundly affecting readiness to adopt health technologies.^63,64^ With respect to this challenge, there is a study^65^ that highlighted that diabetic patients can benefit from alternative energy solutions such as power banks and solar energy to backup QST devices. The use of alternative power sources can increase the readiness of patients to use QST in developing countries. The last challenge was that some elderly participants had technology phobia. Aligned to this finding, one study^66^ argued that the generational digital divide, stereotypes and a lack of tailored solutions exacerbate anxiety among older people. To overcome technophobia and increase the readiness of the elderly to adopt QST, some mitigation strategies are required. There is a study^67^ that found that technological anxiety is a temporary impediment that can be countered by training. Another solution to overcome technophobia is to provide the elderly with family support as uncovered by the results of this study. Moreover, personalised assistance and clear instructions on how to use technology can provide an environment that reduces patient anxiety.

Two main insecurity concerns were uncovered, and they are identity and data theft. They are cyber insecurities that are common amongst online users. The insecurity concerns uncovered in this research align with studies that found that security issues may impede technology readiness.^68,69^ Data theft can result in unauthorised access, identity theft, financial risks, and the commercialisation of health data.^70,71^ Nevertheless, the results of this study uncovered that even though the participants had insecurities, they were optimistic. This suggests that insecurities may not deter the readiness to adopt QST by the users.

## Limitations of the study

The study is limited by a small sample of participants which was drawn from a public hospital in Bulawayo city, Zimbabwe, which may affect the generalisability of the findings to other countries. Even though the results cannot be generalised, they can be transferred to other countries in sub-Saharan Africa and other third world countries, because Zimbabwe is representative of third world countries. Common amongst sub-Saharan African countries is that most have high mobile digital growth which serves as the primary source of internet access and telephone, however, they all have erratic power supply that affects all facets of life. Economically, sub-Saharan countries are third-world countries with noticeable margins between the rich and the poor. Because of poverty in these countries, the governments are responsible for funding the health systems of most of the country's population. This study acknowledges that even though the countries have some similarities, the economic, social and technological environments are not uniform. Whilst this study provides valuable and in-depth insights from the Zimbabwean context, we anticipate that if the study is undertaken in other sub-Saharan African countries or any third world country, the results may vary by small margins due to contextual factors.

Research participants were diabetic patients, thus, insights from medical support professionals could have given insights into the complexity and interplay between medical professionals’ perspectives and how it affects patient readiness to use QST. The voice of medical practitioners is absent in the results of this study.

The researchers acknowledge limitations of self-selection biases.^72,73^ Participants who volunteer to participate in self-sampling studies cannot be representative of the entire population because they can have a biased interest in the phenomena under study.^74,75^ Furthermore, researchers have no control over the selection of participants, which can introduce demographic biases.^72,73^ As witnessed in this study, 70% of the participants indicated that they had attained tertiary education. The lived experiences of those who are educated could not be the same as of those who are uneducated with respect to technology adoption. However, self-selection approach was utilised in the study because the hospital's ethical requirements explicitly stipulated that patient participation in any research was voluntary and the hospital had no obligation of recruiting patients for any research.

## Conclusion

The question answered in this research is, ‘What are the factors that inform the readiness of diabetic patients to adopt QST in developing countries?’. The results of the data analysis found that the five factors that inform the readiness of diabetic patients to adopt self-technology are awareness of technology, optimism, social support, challenges and insecurity.

Future work should focus on a longitudinal study where the diabetics are provided with a QST device to determine how the effects of observed factors determine the adoption of QST in managing diabetes. Incites from the longitudinal study can feed into the development of a model that can act as a guide for the deployment of QST technologies. Furthermore, subsequent research should carry out comparative studies in different developing countries to understand how differences in digital infrastructure, healthcare legislation and cultural norms affect the readiness to adopt QST for managing diabetes.

## Supplemental Material

sj-docx-1-dhj-10.1177_20552076251376286 - Supplemental material for The readiness to use quantified self-technology: A case of diabetic patients from a hospital in Bulawayo, Zimbabwe Supplemental material, sj-docx-1-dhj-10.1177_20552076251376286 for The readiness to use quantified self-technology: A case of diabetic patients from a hospital in Bulawayo, Zimbabwe by Belinda Mutunhu Ndlovu, Baldreck Chipangura and Shawren Singh in DIGITAL HEALTH
