# Supplementary material for: The readiness to use quantified self-technology: A case of diabetic patients from a hospital in Bulawayo, Zimbabwe
Source: Digit Health. 2025 Oct 28;11:20552076251376286. doi: 10.1177/20552076251376286 (PMC12576210; doi:10.1177/20552076251376286)
Supplement: sj-docx-1-dhj-10.1177_20552076251376286 - Supplemental material for The readiness to use quantified self-technology: A case of diabetic patients from a hospital in Bulawayo, Zimbabwe [file sj-docx-1-dhj-10.1177_20552076251376286.docx]

**APPENDIX**

**Supplemental Table 1.** COREQ (COnsolidated criteria for REporting Qualitative research) Checklist

| **Topic** | **Item no.** | **Guide Questions/' Description** | **Reported in the section** |
| --- | --- | --- | --- |
| **Domain 1: Research team and reflexivity** | | | |
| **Personal characteristics** | | | |
| Interviewer/facilitator | 1 | Which author/s conducted the interview or focus group? | The first author, BM, a female PhD student, performed the interviews. The researcher has an MSc in Information Systems and is an experienced researcher in health informatics. She is also employed as a lecturer. This is reported in the methodology section under data collection, Page 5. |
| Credentials | 2 | What were the researcher's credentials? E.g. PhD; MD |  |
| Occupation | 3 | What was their occupation at the time of the study? |  |
| Gender | 4 | Was the researcher male or female? |  |
| Experience and training | 5 | What experience or training did the researcher have? | The interviewer has several years of experience in the field of digital health. |
| **Relationship with participants** | | | |
| Relationship established | 6 | Was a relationship established prior to study commencement? | Participants were invited through a participant invitation form and participant information sheet and contacted by the researcher by telephone, WhatsApp, or email. Rapport was built during the interview. This is highlighted in the Methodology Section under Sampling and Participant Recruitment, Page 3 |
| The participant's knowledge of the interviewer | 7 | What did the participants know about the researcher? e.g. personal goals; reasons for doing the research | The researcher introduced herself as a PhD student and highlighted that the research is for her PhD study. She gave participants the Participant Information Sheet with all these details and the names and emails of the other researchers. She also highlighted that she had received permission from the hospital through a gatekeeper's letter and ethical clearance from her university: Ref (2023/CAES_HREC/1791). Page 5 |
| Interviewer characteristics | 8 | What characteristics were reported about the interviewer/facilitator? e.g. biases, assumptions, reasons and interests in the research topic |  |
| **Domain 2: Study design** | | | |
| \| **Theoretical framework** \| \| --- \| | | | |
| Methodological orientation and theory | 9 | What methodological orientation was stated to underpin the study? e.g. grounded theory; discourse analysis; ethnography; phenomenology; content analysis | This study was a qualitative study leveraging Braun and Clarke's (2006) Thematic Analysis. This is reported in the Methodology Section, under Data Analysis, Page 5 |
| **Participant selection** | | | |
| Sampling | 10 | How were participants selected? e.g. purposive; convenience; consecutive; snowball | Purposive sampling and self-selecting sampling were employed using a predefined inclusion and exclusion criterion highlighted in the methodology section under sampling and participant recruitment. Page 3. |
| Method of approach | 11 | How were participants approached? e.g. face-to-face; telephone; mail; email | The researcher contacted the participants by telephone. However, interviews were held face-to-face at the participants' chosen location. This is reported in the methodology section under sampling and participant recruitment, Page 3. |
| Sample size | 12 | How many participants were in the study? | Initially, 42 participants were recruited, but due to various reasons, under sampling and participant recruitment section, only thirty-five participants were included in the study; Page 4 |
| Non-participation | 13 | How many people refused to participate or drop out? Reasons? |  |
| **Setting** | | | |
| Setting of data collection | 14 | Where was the data collected? e.g. home; clinic; workplace | Interviews were conducted at the participant's preferred location. No other people were present except for the participant and the researcher. This is reported on Page 4 under Data Collection. |
| Presence of non-participants | 15 | Was anyone else present besides the participants and researchers? |  |
| Description of sample | 16 | What are the important characteristics of the sample? e.g. demographic data; date | The sample's demographic characteristics are reported in Table 1, page 5. |
| **Data collection** | | | |
| Interview guide | 17 | Were questions, prompts, and guides provided by the authors? Was it pilot-tested? | An interview guide was developed and piloted. Table 2 Page 5 |
| Repeat interviews | 18 | Were repeat interviews carried out? If yes, how many? | There were no repeat interviews carried out. |
| Audio/visual recording | 19 | Did the research use audio or visual recording to collect the data? | All interviews were audio recorded and transcribed verbatim. The researcher had a reflexive journal, and all debriefing notes were written down. This is reported in the methodology section under data analysis Page 5 |
| Field notes | 20 | Were field notes made during and/or after the interview or focus group? |  |
| Duration | 21 | What was the duration of the interviews or focus group? | Interviews lasted 45 to 60 minutes. This is reported in the Methodology section, Page 5. |
| Data saturation | 22 | Was data saturation discussed? | Data saturation is discussed under data analysis on Page 5 |
| Transcripts returned | 23 | Were transcripts returned to participants for comment and/or correction? | Member checking is reported on Page 4 |
| **Domain 3: analysis and findings** | | | |
| **Data analysis** | | | |
| Number of data coders | 24 | How many data coders coded the data? | Two data coders, BM and BC, independently coded the data. Page 5 |
| Description of the coding tree | 25 | Did the authors provide a description of the coding tree? | A detailed description of the analysis steps is provided in the methodology section. Page 5 under data analysis. |
| Derivation of themes | 26 | Were themes identified in advance or derived from the data? | The identified themes were derived from both the data and in advance. Data analysis is described in detail in the methodology section. Page 5 |
| Software | 27 | What software, if applicable, was used to manage the data? | The qualitative data analysis was facilitated by using ATLAS. ti software.Page 5 |
| Participant checking | 28 | Did participants provide feedback on the findings? | No participants provided feedback on the findings. |
| **Reporting** | | | |
| Quotations presented | 29 | Were participant quotations presented to illustrate the themes/findings? Was each quotation identified? e.g. participant number | Quotations are presented in the results section. Each quotation was indexed using participants' pseudonyms (P1 to P35). The results section presents this. Page 6 to page 10 |
| Data and findings are consistent | 30 | Was there consistency between the data presented and the findings? | We endeavoured to ensure consistency between the data presented and the findings by using quotes to support our interpretations/findings. Please see the results section and quotations provided in italics under the Results section. Page 6 to 10 |
| Clarity of major themes | 31 | Were major themes clearly presented in the findings? | We described our major themes and supporting subthemes. Table 3. Page 6 |
| Clarity of minor themes | 32 | Is there a description of diverse cases or a discussion of minor themes? |  |
